# Imaging the Area of Internal Limiting Membrane Peeling after Macular Hole Surgery

**DOI:** 10.3390/jcm13133938

**Published:** 2024-07-04

**Authors:** Christoph R. Clemens, Justus Obergassel, Peter Heiduschka, Nicole Eter, Florian Alten

**Affiliations:** Department of Ophthalmology, University of Muenster Medical Center, 48149 Muenster, Germany

**Keywords:** macular hole, macular surgery, vitrectomy, internal limiting membrane, en-face optical coherence tomography, confocal scanning laser ophthalmoscopy

## Abstract

**Background**: The aim of this study was to compare en-face optical coherence tomography (OCT) imaging and confocal scanning laser ophthalmoscopy (cSLO) imaging at different wavelengths to identify the internal limiting membrane (ILM) peeling area after primary surgery with vitrectomy and ILM peeling for macular hole (MH). **Methods:** In total, 50 eyes of 50 consecutive patients who underwent primary surgery with vitrectomy and ILM peeling for MH were studied. The true ILM rhexis based on intraoperative color fundus photography was compared to the presumed ILM rhexis identified by a blinded examiner using en-face OCT imaging and cSLO images at various wavelengths. To calculate the fraction of overlap (FoO), the common intersecting area and the total of both areas were measured. **Results:** The FoO for the measured areas was 0.93 ± 0.03 for en-face OCT, 0.76 ± 0.06 for blue reflectance (BR; 488 nm), 0.71 ± 0.09 for green reflectance (GR; 514 nm), 0.56 ± 0.07 for infrared reflectance (IR; 815 nm) and 0.73 ± 0.06 for multispectral (MS). The FoO in the en-face OCT group was significantly higher than in all other groups, whereas the FoO in the IR group was significantly lower compared to all other groups. No significant differences were observed in FoO among the MS, BR, and GR groups. In en-face OCT, there was no significant change in the ILM peeled area measured intraoperatively and postoperatively (8.37 ± 3.01 vs. 8.24 ± 2.81 mm^2^; *p* = 0.8145). Nasal-inferior foveal displacement was observed in 38 eyes (76%). **Conclusions:** En-face OCT imaging demonstrates reliable postoperative visualization of the ILM peeled area. Although the size of the ILM peeling remains stable after one month, our findings indicate a notable inferior-nasal shift of the overall ILM peeling area towards the optic disc.

## 1. Introduction

Substantial progress has been made in macular hole (MH) surgery in recent years. After Kelly and Wendel reported the first series of patients to undergo vitreous surgery for idiopathic MH in 1991, Eckardt et al. revolutionized surgery for MH closure introducing the internal limiting membrane (ILM) peeling in 1997 [1,2]. A subsequent milestone was the introduction of the inverted ILM flap technique by Michalewska and colleagues, which further improved the anatomical closure rate [3]. Following the original description, a number of variations of the ILM flap technique have been described [4,5,6,7,8,9]. The ILM is formed by the basement membrane of the Müller cells, representing the inner surface of the retina [10]. Mechanically, it is distinctly stronger and stiffer than the cellular layers of the retina and accounts for 50% of retinal tensile strength [11,12]. This indicates the central role of the ILM in the biomechanical integrity of the retina and that manipulation of this layer during ILM peeling considerably impacts retinal biomechanics. 

Clinical research in recent years has shown that the size and localization of the ILM peeling are the most important considerations for the surgeon that determine the anatomical and functional success of the procedure in terms of closure rate, metamorphopsia, foveal displacement, asymmetric elongation of the foveal tissue, and the extent of inner retinal dimpling (IRD) [13,14,15]. A large peeling area has the advantage of less metamorphopsia; a small peeling area, on the other hand, benefits from less foveal displacement and fewer microscotomas due to IRD [16,17,18,19]. The ideal approach would be a preoperative planning of the peeling area, which individually offers the best trade-off between those aspects. To achieve this goal, however, many clinical studies are still required, for which an accurate and reliable method for determining the ILM peeling area is essential. 

In the past, different approaches of visualizing the area of ILM peeling postoperatively have been reported. Weinberger et al. described indocyanine green (ICG) fluorescence originating from the residual ILM that may be detected postoperatively by infrared fundus imaging after MH surgery using ICG for ILM staining [20,21]. Tadayoni et al. presented, for the first time, a blue filter fundus photograph showing distinctly the boundaries of the ILM removal [14]. In 2005, Miura et al. described the delineation of the ILM peeling area using confocal scanning laser ophthalmoscopy (cSLO) imaging at wavelengths of 488 nm and 514 nm [22]. Since the first prototype en-face OCT images were taken over twenty years ago, technical progress has been made continuously. Today, recent advances in rapid scanning speeds and high-density volume data sets allow for digital reconstruction of retinal layers in the en-face plane, allowing detailed analysis of the retina at specified segmentation levels [23]. Numerous authors have reported detailed visualization of various pathological processes at the level of the ILM using this imaging technique [24,25,26]. The extent to which the ILM peeling area measured postoperatively actually corresponds to the exact intraoperative area remains unclear in view of foveal displacement, imaging artefacts, and postoperative migration, as Sahoo et al. recently highlighted [26].

Therefore, this study aims to compare postoperative en-face OCT imaging with multispectral cSLO imaging (MS) using various wavelengths (488 nm for blue, 514 nm for green, and 815 nm for infrared) to delineate the extent of ILM peeling in MH surgery, contrasting it with the intraoperative ILM peeling area. The different wavelengths of MS images are utilized to visualize distinct retinal layers: blue light emphasizes the inner retinal layers, green light penetrates deeper, and infrared light captures details from the outer retinal layers.

## 2. Materials and Methods

Patients with an idiopathic full-thickness MH with an elevated retinal cuff and no signs of epiretinal gliosis were retrospectively recruited at the Department of Ophthalmology at the University of Muenster, Germany, between July 2020 and September 2023. Eyes were not eligible if any signs of retinal pathology other than a full-thickness MF were observed in funduscopy, SD-OCT, or cSLO. All patients underwent comprehensive ophthalmologic examinations, including the measurement of best-corrected visual acuity (BCVA). 

Patients underwent surgery using the internal limiting membrane (ILM) flap technique [3]. A standard three-port 23-gauge pars plana vitrectomy was performed. When necessary, a posterior hyaloid separation was induced, and the posterior hyaloid was completely removed. To facilitate ILM visualization, ILM-blue dye (DORC) was injected onto the macular region, staining the ILM for approximately 30 s. Using ILM forceps (23G, Advanced DSP Tip, Alcon Laboratories, Inc., Fort Worth, TX, USA), the ILM was peeled in a circular manner for approximately two disk diameters around the MH, creating a temporal flap. This flap was then inverted and positioned over the MH. A balanced saline solution–air exchange was performed, followed by the injection of expansile gas (C3F8 16%) at the end of the surgery. Phakic eyes underwent combined phacoemulsification with intraocular lens implantation and vitrectomy. Postoperatively, patients were maintained in a face-down position overnight and were advised to remain in a prone position for three days following the surgery.

Intraoperatively, the surgeon recorded a color fundus photography (CFP) after ILM staining and ILM peeling, serving as ground truth for the localization and size of the ILM peeled area. Multimodal imaging was performed preoperatively and four to six weeks postoperatively (Heidelberg Retina Angiograph—OCT; Heidelberg Engineering, Heidelberg, Germany). Spectral-domain OCT of the posterior pole was acquired in the same session as multispectral (MS) cSLO imaging containing three separate cSLO images of 488 nm (blue [BR]), 518 nm (green [GR]), and 820 nm (infrared [IR]). For en-face OCT imaging, we used a custom slab to visualize the ILM peeled area. The area was quantified using the measurement tool provided by the OCT software (Heidelberg Eye Explorer 1.12.1.0). For area quantification in color fundus photography, the pixel counting function in the histogram window of Adobe Photoshop (CS6 Extended) was used.

Two retinal specialists (C.C. and F.A.) conducted a subjective evaluation of the discernibility of the ILM peeling margins across all imaging modalities. This evaluation utilized criteria established by Miura et al., which categorized the margin clarity as follows: excellent (very clear), very good (clear), moderate (identifiable but blurred), poor (barely identifiable), and very poor (not identifiable) [22].

**Figure 1:**
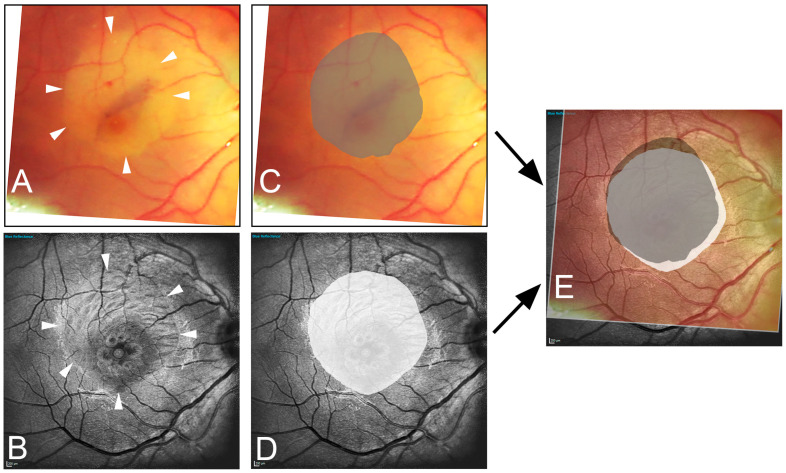
Illustration of the image data acquisition process. The edges of the peeled area are marked in the intraoperative image (**A**) (arrowhead) and in the one-month postoperative blue reflection image (**B**) (arrowhead). Area delineation was performed by the surgeon (dark gray) (**C**) and the blinded examiner (white) (**D**). Subsequently, the images are superimposed (**E**), resulting in the fraction of overlap (light gray area). Note the inferior-nasal displacement toward the optic nerve head (white area).

In a second analysis, intraoperatively recorded CFP images after ILM staining and peeling were transformed to align with postoperative MS cSLO and en-face OCT images using Adobe Photoshop, based on the retinal blood vessel patterns for orientation. This transformation included adjustments for scaling, rotation, and distortion as necessary. After overlaying images (CFP/MS cSLO and CFP/en-face OCT), the area of peeled ILM was delineated in the postoperative images by the surgeon determining the true size and localization of ILM peeled area. A blinded, independent examiner (FA) marked the presumed border of the ILM rhexis in each en-face OCT, multispectral, BR, GR, and IR image. Subsequently, the respective images with the true ILM rhexis based on intraoperative imaging and the presumed ILM rhexis marked by the blinded examiner were compared. After the overlay of corresponding images, the common intersecting area and the sum of both areas were determined using the pixel-counting function of the histogram window in Adobe Photoshop (Figure 1). The fraction of overlap (FoO) was calculated by dividing the former by the latter:

(1) $$fraction\;of\;overlap=\frac{common\;intersecting\;area}{sum\;of\;marked\;areas}$$

By forming a fraction of the matching areas in the numerator and the sum of marked areas in the denominator, a measure is obtained that reflects the accuracy of the ILM rhexis identification in the respective modality. The values are between 0 and 1. The closer the value is to 1, the higher the accuracy. A perfect value of 1 would mean that all selected pixels of the blinded examiner match the pixels of the real ILM peeled area, while none of the selected pixels were falsely marked. 

The FoO data were analyzed using GraphPad Prism 10 software. Normality testing was conducted using multiple tests: D’Agostino and Pearson, Anderson–Darling, Shapiro–Wilk, and Kolmogorov–Smirnov. The data did not pass normality tests in 11 out of 20 cases, and the normal QQ plot indicated a skewed distribution. Therefore, we proceeded with the Kruskal–Wallis test followed by Dunn’s multiple comparison test to assess the statistical differences between groups. In contrast, en-face OCT measurements of intra- and postoperative ILM peeled areas showed a Gaussian distribution and were analyzed using an unpaired *t*-test.

## 3. Results

Due to imaging artifacts, 50 out of a total of 61 eyes from 59 patients (34 women) were ultimately analyzed. The patients were in an age group ranging from 64 to 74 years (mean ± SD = 67.5 years ± 3.4). In total, 31 eyes were phakic; 19 were pseudophakic. The median preoperative logMAR BCVA was 0.8 ± 0.2 (Snellen equivalent 20/125). The aperture size of the MH as measured from spectral domain OCT images ranged from 325 μm to 1112 μm, with a mean aperture size of 631 μm. MH larger than 400 μm were present in 43 eyes. All patients were first re-examined in a mean follow-up period of 32.5 days (range: 25 to 43 days). In total, 46 of the 50 eyes (92%) had a closed MH at the first follow-up review. In the closed MH group, the median BCVA improved from 0.7 logMAR units preoperatively to 0.1 logMAR units (Snellen equivalent 20/25) postoperatively. 

**Figure 2:**
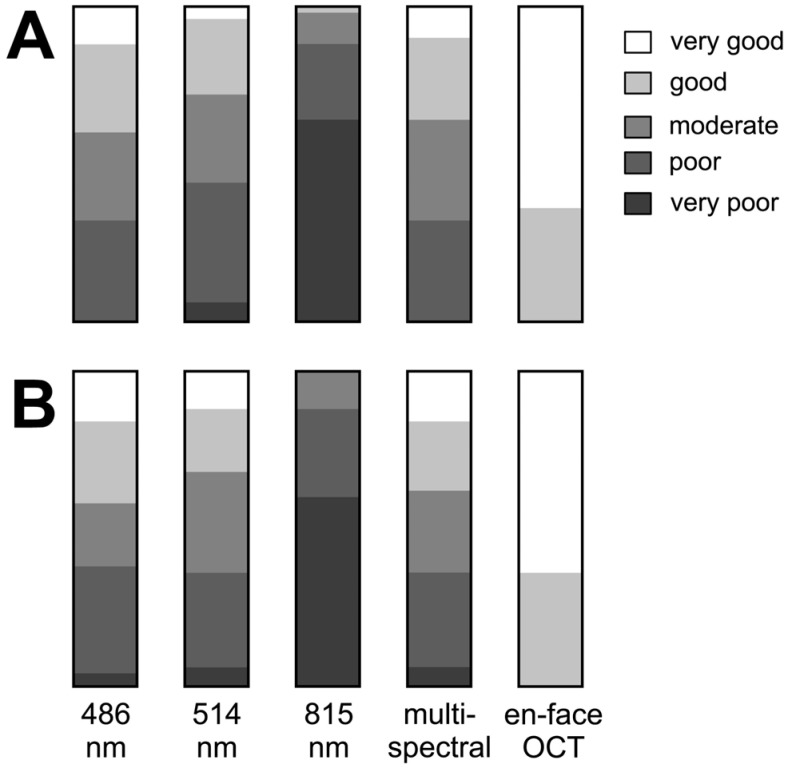
Results of subjectively grading the margin of internal limiting membrane peeling ((**A**) by C.C. and (**B**) by F.A.). The bar graphs show results for each grader for each image type: cSLO images at 486 nm, 514 nm, 815 nm, multispectral, and en-face OCT.

Figure 2 illustrates the varying degree of subjective visualization quality of the rhexis edge in the different imaging modalities. 

**Figure 3:**
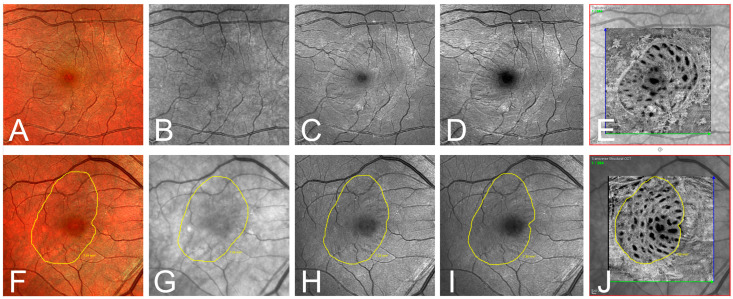
(**A**–**E**) Exemplary one-month postoperative imaging after macular hole surgery with internal limiting membrane peeling. Confocal scanning laser ophthalmoscopy (cSLO) images: (**A**) multispectral (MS), (**B**) infrared (IR), (**C**) green reflectance (GR), (**D**) blue reflectance (BR), and (**E**) en-face optical coherence tomography (OCT). Notice the clear identification of the ILM rhexis in the en-face OCT, and the rather poor visibility in the IR and GR images. (**F**–**J**) Exemplary presentation of another patient one month postoperatively. Illustration of the ILM-border (yellow line) defined by the grader in the different modalities: (**F**) MS, (**G**) IR, (**H**) GR, (**I**) BR, and (**J**) en-face OCT.

**Figure 4:**
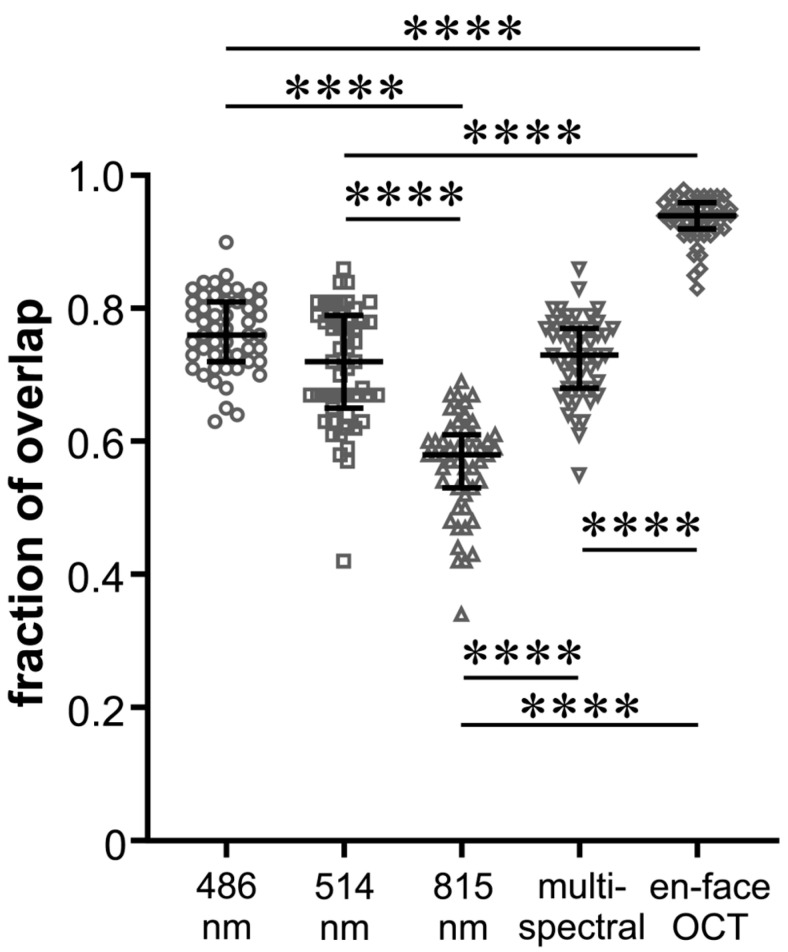
Beeswarm plot of individual values of fraction of overlap of the peeling areas for the five different imaging modalities as indicated. The black lines show the median and the interquartile range for each group; **** *p* < 0.0001.

FoO of the measured areas was 0.76 ± 0.06 (BR), 0.71 ± 0.09 (GR), 0.56 ± 0.07 (IR), and 0.73 ± 0.06 (MC) with the highest FoO value in the en-face OCT group (0.93 ± 0.03) (Figure 3). In Figure 4, it can be seen that the values of the FoO were significantly smaller in the IR group compared to all other groups (*p* < 0.0001 for all groups vs. IR). There was no significant difference among the MC group, BR group, and GR group (BR vs. GR 0.313; BR vs. MC 0.892; GR vs. MS > 0.999).

In en-face OCT, there was no significant change in the ILM peeled area measured intraoperatively and postoperatively (8.37 ± 3.01 vs. 8.24 ± 2.81 mm^2^; *p* = 0.8145). In 38 eyes (76%), a nasal or nasal-inferior foveal displacement was observed (Figure 1E).

## 4. Discussion

In this study, we evaluated whether en-face OCT and cSLO imaging at different wavelengths would allow us to reliably identify the borders of ILM rhexis after primary surgery with vitrectomy and ILM peeling for MH. In brief, we found that en-face OCT imaging provides the most reliable postoperative visualization of the ILM peeled area. While the ILM peeling size is stable after one month, the overall ILM peeling area has shifted inferior-nasally towards the optic disc. 

The ILM is the major reflector at the retinal surface and significantly contributes to retinal images at short wavelengths [27,28,29]. In 2005, Miura et al. compared the effectiveness of visualizing the border of ILM peeling with spectral SLO imaging, CFP, and red-free fundus photography, reporting that the margin of ILM peeling was best visible in SLO imaging at 488 nm and 514 nm [22]. Their results are in line with our findings regarding cSLO wavelengths. As expected, the identification of the ILM rhexis edge was best in the BR and in the MS modality because the anatomical location of the ILM favors the absorption of shorter wavelengths. Most of the absorption of blue light occurs in the inner part of the retina, the green wavelengths penetrate slightly deeper, and most of the information in the infrared image derives from the outer retinal layers and choroids. Notably, Miura et al. reported that the edge of the ILM peeling was clearly or very clearly recognizable in only about 50% of patients, while in the other 50%, the ILM edge was blurred, barely recognizable, or not recognizable. In our study, the results of the subjective identification of the ILM rhexis edge in cSLO images of different wavelengths were slightly better overall compared to Miura et al., most likely due to improvements in the cSLO devices since their study in 2005. Compared to all cSLO wavelengths, en-face OCT proved superior in the subjective identification of the ILM rhexis edge. The quantitative analysis of the ILM peeling area using FoO showed a similar picture with very good values for en-face OCT and only mediocre values for BR, GR, and MS cSLO. Overall, the identification of the ILM rhexis edge in cSLO is unsatisfactory and not reliable for evaluation in clinical studies, whereas en-face OCT allows a clear delineation of the ILM peeled area. The question remains whether the area clearly visualized by postoperative en-face OCT actually corresponds to the area of intraoperative ILM peeling. Two variables are crucial here: postoperative foveal displacement and postoperative cell proliferation at the ILM rhexis edge.

One unfavorable effect of ILM peeling is the foveal displacement of the retina towards the optic nerve head, as was first shown by Yoshikawa et al. [30]. Shortly thereafter, Kawano et al. and Ishida et al. observed this phenomenon in MH patients and attributed it to the increased flexibility of the central retina after ILM peeling and the constant traction of the optic nerve head [31,32]. Studies described the extent of the mean foveal displacement as approximately 50 and 200 µm, respectively [33,34]. It has been hypothesized that ILM peeling leads to the shrinkage of the retinal nerve fiber (RNF) at the site where the ILM was removed as RNF consists predominantly of microtubules and their depolymerization causes axons to shrink [31,35]. Notably, the degree of foveal displacement correlates with the extent of ILM peeling [16,34]. Taken together, the localization of the ILM peeled area postoperatively, despite exact visualization, does not correspond exactly to the intraoperative localization. This is also reflected in our results. Despite very good visualization of the ILM rhexis edge in the en-face OCT image, the postoperative nasal and inferior displacement results in a slightly inaccurate match of the surfaces, yielding only an FoO of 0.93 (Figure 4). 

Nakamura et al. studied the ultrastructure of the vitreoretinal interface after removal of the ILM in primates using electron microscopy [36,37]. Interestingly, they found that after three months, reactive glial cells such as Müller cells and astrocytes begin to cover the edge of the ILM and, in the further course, also covered almost the entire ILM peeled area. There are no comparable histological studies in humans. Nonetheless, the available clinical studies in humans suggest that the gliosis after ILM peeling is by far not as severe as in the mentioned histological study on monkey eyes [38]. Ishida et al. reported that ILM peeling promotes the proliferation of preretinal abnormal tissues (PATs) in eyes with MH using en-face OCT. Outside the area of ILM peeling, postoperative PAT expanded from the edge of ILM peeling toward the periphery as early as 2 weeks postoperatively, whereas inside the area of ILM peeling, PAT was only observed in 15% [24]. Our results show that the absolute area of ILM peeling measured intraoperatively and four weeks postoperatively is approximately the same, suggesting that cell proliferation at the ILM rhexis edge has a minimal impact on the measurement of the ILM peeling area. 

Intraoperative photo documentation of the ILM peeled area offers the advantage of being unaffected by postoperative foveal displacement or postoperative cell proliferation [39,40]. However, potential inaccuracies in translating intraoperative photographs to the postoperative imaging modality may pose a challenge. 

Doubtlessly, ILM peeling in MH surgery is beneficial in increasing the rate of closure and decreasing the rate of MH reopening [41]. Despite such benefits, we must face its structural and functional consequences that are so far not well understood like microscotoma, decrease in retinal sensitivity, reduction in the focal electroretinogram, and the development of IRD [15,19,42,43]. So far, there is no consensus regarding the optimal extent and localization of the ILM peeled area [44]. In this context, a clear identification of the area of ILM peeling is crucial to evaluate the various types of retinal damage. In view of these negative effects of ILM peeling, “as much as necessary and as little as possible” seems to be the right approach. Furthermore, the localization of ILM peeling appears to be important, as discussed by numerous authors [45,46]. Overall, many aspects in this field are still unclear, so further clinical research is needed to continue to improve the results. Certainly, the best possible imaging method for quantifying and localizing the ILM peeling area is essential for that purpose. En-face optical coherence tomography (OCT) represents a valuable tool in the early postoperative period following macular hole surgery, offering several critical advantages. Firstly, it facilitates a meticulous evaluation of the extent and dimensions of the peeled internal limiting membrane (ILM), providing clinicians with precise quantitative data regarding this crucial surgical aspect. Moreover, en-face OCT allows for a detailed analysis of retinal displacement, enabling clinicians to assess the exact degree of displacement of retinal layers and potential changes in macular morphology post-surgery. These capabilities enhance the ability to monitor surgical outcomes comprehensively and make informed decisions regarding further management or intervention, thereby improving overall patient care and visual prognosis. A vision for the future could involve a preoperative planning of the peeling area, which individually offers the best trade-off between the positive and negative aspects of ILM peeling. With the increasing digitalization of surgical microscopes, an intraoperative display of the preoperatively determined peeling area for each individual patient also seems conceivable.

Apart from the retrospective study design, major limitations were the small sample size and the short postoperative follow-up.

In conclusion, en-face OCT imaging demonstrates reliable postoperative visualization of the ILM peeled area. Although the size of the ILM peeling remains stable after one month, our findings indicate a notable inferior-nasal shift of the overall ILM peeling area towards the optic disc.

## Data Availability

Data available upon reasonable request.
